# 1160. Comparative Effectiveness of Pneumococcal Vaccination Strategies to Prevent Invasive Pneumococcal Disease: A Cohort Study at the Veterans Health Administration

**DOI:** 10.1093/ofid/ofad500.1000

**Published:** 2023-11-27

**Authors:** Michihiko Goto, Satoshi Kakiuchi, Eli N Perencevich, Shinya Hasegawa, Michael Jones

**Affiliations:** University of Iowa/Iowa City VAMC, Iowa City, Iowa; Nagasaki University School of Medicine, Nagasaki, Nagasaki, Japan; University of Iowa/Iowa City VAMC, Iowa City, Iowa; University of Iowa Carver College of Medicine, Iowa City, Iowa; University of Iowa College of Liberal Arts and Sciences, Iowa City, Iowa

## Abstract

**Background:**

The direct impact of a combined vaccination strategy pneumococcal 13-valent conjugate vaccine (PCV13) and 23-valent polysaccharide vaccine (PPSV23) for adults in preventing pneumococcal infections has yet to be thoroughly investigated. We aimed to estimate the comparative effectiveness of preventing the most severe form of pneumococcal infections, invasive pneumococcal disease (IPD), by comparing the risk of IPD stratified by the use of PCV13, PPSV23, and their combinations.

**Methods:**

We included all patients who were 65 years or older, had established primary care within the Veterans Health Administration (VHA) between 2005 and 2021, and had not received any prior pneumococcal vaccination. We measured time-to-event from cohort enrollment to the onset of culture-proven IPD, considering death a competing risk event, and constructed a Cox regression model to estimate cause-specific hazards. PCV13 only, PPSV23 only, and PCV13 + PPSV23 were incorporated into the model as a time-dependent exposure variable, and we estimated hazard ratios (HRs) associated with them. We also considered a waning effect by incorporating time-by-vaccine interaction terms in the model to estimate continuously changing time-dependent hazards.

**Results:**

5,069,917 patients were enrolled in the cohort (male: 96.5%; median age: 72 [IQR: 65-79]). 1,217,349 patients received at least one dose of pneumococcal vaccine during the study period. The overall incidence rate of IPD was 9.1 per 100,000 patient-years. In crude analysis, all pneumococcal vaccines were associated with lower HRs for IPD, and there was an additive benefit of PCV13 and PPSV23 (Tables 1 and 2). In multivariate analysis, associations between PCV13 alone and PPSV23 alone were no longer statistically significant after adjusting for age and comorbidities, but PCV13 + PPSV23 was significantly associated with lower HR for IPD. No time-by-vaccine interaction term was significant.

**Figure:**
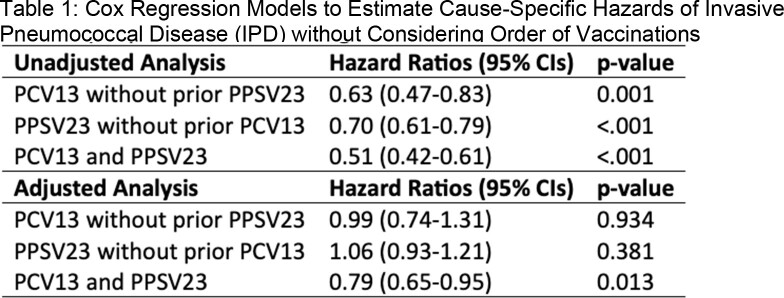

**Figure:**
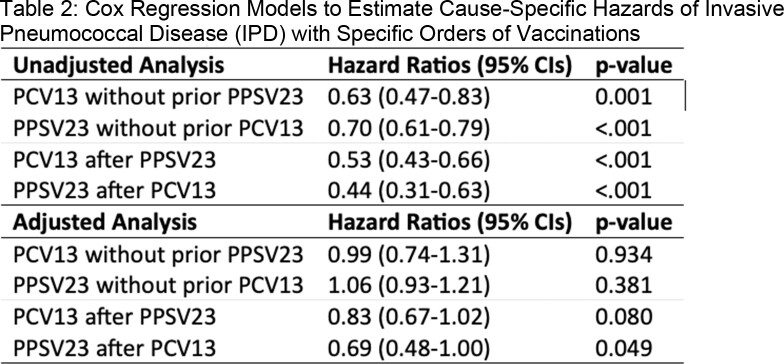

**Conclusion:**

In this large cohort study at the VHA, neither vaccine by itself appeared to lower the risk of IPD, but the combination of PCV13 and PPSV23 was associated with a significantly lower risk. In addition, we did not observe evidence of a waning effect to prevent IPD. Further studies are needed to evaluate the effect of newer pneumococcal vaccines.

**Disclosures:**

**Michihiko Goto, MD MSCI**, Merck & Co.: Grant/Research Support

